# The Potential Therapeutic Application of Simvastatin for Brain Complications and Mechanisms of Action

**DOI:** 10.3390/ph16070914

**Published:** 2023-06-22

**Authors:** Yen My Vuu, Ashraf Kadar Shahib, Mojgan Rastegar

**Affiliations:** Department of Biochemistry and Medical Genetics, Max Rady College of Medicine, Rady Faculty of Health Sciences, University of Manitoba, Winnipeg, MB R3E 0J9, Canada

**Keywords:** cholesterol, medulloblastoma, Alzheimer’s disease, Parkinson’s disease, Huntington’s disease, ApoE, CYP46A1, LDLR, Hh family, lipid rafts

## Abstract

Statins are common drugs that are clinically used to reduce elevated plasma cholesterol levels. Based on their solubility, statins are considered to be either hydrophilic or lipophilic. Amongst them, simvastatin has the highest lipophilicity to facilitate its ability to cross the blood-brain barrier. Recent studies have suggested that simvastatin could be a promising therapeutic option for different brain complications and diseases ranging from brain tumors (i.e., medulloblastoma and glioblastoma) to neurological disorders (i.e., Alzheimer’s disease, Parkinson’s disease, and Huntington’s disease). Specific mechanisms of disease amelioration, however, are still unclear. Independent studies suggest that simvastatin may reduce the risk of developing certain neurodegenerative disorders. Meanwhile, other studies point towards inducing cell death in brain tumor cell lines. In this review, we outline the potential therapeutic effects of simvastatin on brain complications and review the clinically relevant molecular mechanisms in different cases.

## 1. Introduction

The brain is an extremely complex organ in the human body. There has been an intensive research interest towards brain development, function, and anatomy since early scientific reports about this highly complex organ [1,2]. In our modern scientific era, neuroscience has emerged as an essential field of study. This is because the brain spearheads the regulation of many activities in our body that range from cognitive function, movement, breathing, food digestion/absorption, sleep cycles, and senses, to emotion. In addition, brain-related diseases have been the center of attention, as they involve major dysfunction of one or multiple functions of our body, and many of these diseases have no available cure.

The molecular mechanisms behind the brain’s regulatory roles and possible complications largely rely on dynamic metabolic events, such as glucose/lipid metabolism, oxygen consumption, and utilization of amino acids. Amongst these, abnormal levels of brain cholesterol are commonly associated with neurological disorders. Cholesterol is a type of lipid that is abundantly found across the body, particularly in the central nervous system (CNS). In the brain, cholesterol is involved in a wide range of cellular components and processes. For instance, it is an essential component of myelin (the insulating layer around neurons), an important factor to promote synaptogenesis (communicative junctions between neurons), required for neurite outgrowth (projections of neurons), and others [3,4,5].

**Figure 1:**
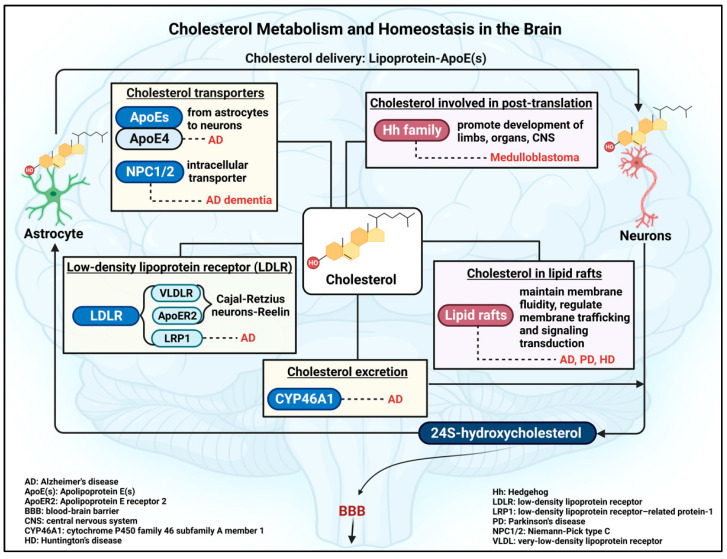
Cholesterol metabolism and homeostasis in the brain. Brain cholesterol is mainly synthesized in the astrocytes and transported into neurons through lipoprotein-ApoEs. Cholesterol is tightly regulated through many factors, such as ApoEs, LDLR, NPC1/2, and CYP46A1. The ApoE4 isoform is highly prevalent in Alzheimer’s disease (AD) pathology, while LRP1 may be a key factor in reducing the risk of AD. The abnormal level of NPC1/2, a factor involved in intracellular cholesterol transporters, is related to AD dementia. In addition, the accumulation of cholesterol in neurons caused by impaired CYP46A1 activity is associated with AD. Beyond its general roles, cholesterol also contributes to post-translational modification and an essential composition of lipid rafts. Disturbed Shh signaling can lead to medulloblastoma brain tumor; meanwhile, dampened lipid rafts may be linked to multiple diseases, such as AD, Parkinson’s disease (PD), and Huntington’s disease (HD). Yellow rectangles indicate upstream factors of brain cholesterol homeostasis. Pink rectangles show the downstream biological role of cholesterol, particularly in neurons. Information obtained from [8,14,15,16,17,18,19,20,21,22,23,24,25,26,27,28,29,30,31,32,33,34,35,36,37,38,39,40]. This Figure is created with BioRender.com.

Although the human brain only accounts for about 2% of our body weight (average 1336 g in adult males and about 1198 g in adult females), it holds around 23% of the total body’s cholesterol [6,7]. Brain cholesterol is locally synthesized and requires balancing to maintain the brain function [6]. This key metabolic feature was further explored in studies that noted a strong association between abnormal brain cholesterol metabolism and neurological diseases, such as Alzheimer’s disease (AD), Parkinson’s disease (PD), and Huntington’s disease (HD) [8,9,10] (Figure 1). Hence, the level of brain cholesterol must be stably maintained to sustain normal brain function. In this regard, statins, particularly simvastatin, may effectively influence brain cholesterol levels due to their ability to cross the blood-brain barrier (BBB). Statins have been suggested as potential therapeutic drugs for neurological diseases by balancing cholesterol metabolism [11,12,13]. Therefore, the purpose of this review is to provide a brief overview of the potential therapeutic role of statins and the associated mechanisms, focusing on simvastatin as a candidate therapeutic drug for brain complications.

## 2. Cholesterol Metabolism and Homeostasis in the Human Body

Cholesterol is an important lipid in the body that fulfills many physiological functions. For instance, it is an indispensable component of the cellular membrane and a precursor of many biomolecules, such as steroid hormones, vitamin D, and bile acids. Though it is involved in different cellular functions, cholesterol levels are tightly regulated, and abnormal levels may lead to serious health issues. A recent study stressed that an abnormal accumulation of cholesterol-containing lipoproteins, such as low-density lipoprotein (LDL) cholesterol, has been identified as one of the major causes of stroke, myocardial infarction, and peripheral arterial diseases. The study also indicated the importance of early preventive strategies, including a healthy lifestyle and the use of cholesterol-lowering drugs, such as statins. Together, these may prevent further incidence of cholesterol-associated health complications in people with high risks of cardiovascular diseases [41]. Hence, it is important to maintain cholesterol homeostasis in the body.

### 2.1. Peripheral Tissues: Protective Mechanisms for Balancing Cholesterol

In peripheral tissues, cholesterol is obtained from two different sources that includes the dietary cholesterol and its synthesis in the liver. Cholesterol synthesis takes place through the mevalonate pathway with a series of enzymatic reactions. A crucial regulatory reaction is performed by the rate-limiting enzyme “3-hydroxy-3-methyl-glutaryl-coenzyme A (HMG-CoA)” reductase, which converts HMG-CoA to mevalonate, a fundamental precursor for many biometabolites, including cholesterol. Of note, cholesterol pooling through synthesis in the liver is also regulated by dietary cholesterol [42]. Upon initial intake of dietary lipids, chylomicron, a dietary lipid-carrier lipoprotein created by intestinal enterocytes [43], functions as the main lipoprotein that carries dietary triglycerides/lipid content to different tissues in the body. Then, the chylomicron remnants containing cholesterol are cleared from the circulation by the liver [44]. When the liver absorbs dietary cholesterol, de novo cholesterol synthesis is decreased as a compensatory mechanism.

In the liver, cholesterol can be used for multiple biological purposes, such as incorporation into bile acids, helping to facilitate dietary lipid digestion/absorption and lipid/cholesterol excretion [45]. Consequently, the liver acts as the main site that maintains cholesterol homeostasis. A study showed that, to balance cholesterol levels in the body, the liver may synthesize about 800 mg of cholesterol daily, which approximately equals the amount of cholesterol excreted *via* bile per day [46]. Moreover, cholesterol and other types of lipids from the liver can be carried by lipoproteins across the bloodstream to different targeted tissues. As such, cholesterol is trapped inside the plasma lipoproteins, which include very-low-density lipoprotein (VLDL), intermediate-density lipoprotein (IDL), LDL, and high-density lipoprotein (HDL). However, the content of lipid/cholesterol carried by these lipoproteins is varied. While VLDL contains the largest amount of triglycerides, LDL carries the highest level of cholesterol in the bloodstream [47]. Lipoproteins deliver lipids/cholesterol to the targeted cells, where certain lipoprotein receptors on cellular surfaces promote the uptake of lipoproteins through receptor-mediated endocytosis [48]. In cells, lipoproteins are hydrolyzed by lysosomal enzymes to release cholesteryl esters and other lipid-derived metabolites, such as cholesterol, triglycerides, and fatty acids [49,50]. Subsequently, cholesterol is used as a substrate for multiple biosynthetic pathways, such as the production of vitamin D and steroid hormones, as well as components of plasma membranes. Therefore, impairment of cholesterol-related carriers, receptors, and cholesterol-synthesis-linked enzymes could lead to abnormal cholesterol levels.

Defects in the hepatic LDL receptor (LDLR) are the main cause of abnormal cholesterol levels in the plasma. The role of LDLR in balancing plasma cholesterol levels was first reported by Brown and Goldstein in 1986, when they studied the physiological mechanism of a genetic disease called familial hypercholesterolemia (FH) [51]. This is an inherited disease that causes significantly elevated LDL cholesterol in the blood due to the functional defects of cholesterol homeostasis-associated factors, such as the blood LDLR and apolipoprotein B (ApoB) [52]. Patients with FH exhibit very high cholesterol levels in their plasma, which may later increase the risk of premature cardiovascular death [53]. Interestingly, Goldstein and Brown found that the activity of HMG-CoA reductase in homozygous FH cells was significantly elevated from 50-fold to 100-fold compared to the baseline. Moreover, among the two main lipoproteins, LDL and HDL, the inhibition of HMG-CoA reductase activity was exclusively associated with LDL [51,54]. Hence, it is suggested that activation of LDLR can reduce HMG-CoA reductase activity as a regulatory feedback response towards plasma LDL cholesterol [54]. Remarkably, evidence indicates that dysfunctional LDLR in FH patients may increase the risk of cognitive impairment and cerebrovascular disorders [55,56]. Therefore, it is suggested that abnormal cholesterol affects not only peripheral tissues but also the CNS.

### 2.2. Brain Cholesterol: De Novo Cholesterol Synthesis and Homeostatic Mechanism

On the contrary to the extrahepatic tissues, brain cholesterol is exclusively synthesized from local de novo synthesis, which takes place in both neurons and astrocytes. During neurodevelopment, neurons produce a higher level of cholesterol than astrocytes, suggesting that the developing neurons are dependent on endogenous cholesterol synthesis [57,58]. However, in adult human brains, astrocytes mainly produce cholesterol in small amounts, as the elimination half-life of the cholesterol content of the human brain is estimated to be around 5 years [14]. As such, neuronal cholesterol relies on the transportation from astrocytes to neurons *via* cholesterol-mediated apolipoprotein E (ApoE) [15,16]. Interestingly, amongst three main isoforms, ApoE2, ApoE3, and ApoE4, researchers found that ApoE4 is strongly linked to the late onset of AD compared to other polymorphic forms of ApoE alleles [17,18]. ApoE is predominantly synthesized in the liver, astrocytes, and microglia [17,59]. ApoE binds to certain members of the LDLR family located on the neuronal membrane, such as the very-low-density-lipoprotein receptor (VLDLR), low-density lipoprotein receptor-related protein-1 (LRP1), and apolipoprotein E receptor 2 (ApoER2)*,* to conduct lipoprotein-ApoE endocytosis [37,60,61].

Amongst LDLR members, LRP1 has a higher endocytosis rate compared to VLDLR and ApoER2, implying a distinct endocytic efficiency of LDLR members [62]. After entering the cells, the lipoprotein-ApoE-LDLR complex is dissociated by the acidic environment and enzymatic reactions within the lysosomes, which ultimately leads to the release of lipid/cholesterol [63]. Extensive studies propose the important role of LDLR in maintaining brain cholesterol homeostasis and brain function. For example, the expression of the neuronal LRP1 is strongly required for synaptic transmission. A study showed the interaction between LRP1 and postsynaptic density protein 95 (PSD-95), which is a postsynaptic scaffolding protein in excitatory neurons and extensively important for healthy motor neurons [64]. Therefore, it is believed that abnormal levels of LDLR may impair brain function.

A 2013 study showed that LRP1 critically contributed to neuronal amyloid-β (Aβ) clearance [65]. Insoluble Aβ is a neuronal toxin that may result in disrupted neuronal membrane potential, synaptic loss, and neuronal death [66]. The significantly increased Aβ accumulation in the brain was found in the neuronal *Lrp1* knockout mice. The authors suggested that neurons were not only producing Aβ but also eliminating Aβ through the receptor-mediated endocytosis of LRP1 in neurons [65]. Similarly, another study showed the clearance role of LRP1 in neuronal Aβ42, which helps in preventing neuronal cytotoxicity and is strongly associated with AD pathology. As such, LRP1 endocytosis helps promote the uptake of Aβ42 to neuronal lysosomes, where the toxic Aβ42 is hydrolyzed [37]. In addition to LRP1, the other VLDLR and ApoER2 receptors are also important to the physiology and function of the brain. A 1999 study discovered the essential binding of Reelin on VLDLR and ApoER2 to promote layer formation in the neocortex, cerebellum, and hippocampus [19]. The glycoprotein Reelin is produced by Cajal-Retzius neurons, which are cells located in the marginal zone of the cortex during early brain development. In this regard, Reelin signaling distributes to migrating neurons and later involves in the formation of cortical cell layers [20]. Reelin binding to VLDLR and ApoER2 activates tyrosine phosphorylation of disabled-1 (DAB1), which functions as an adapter protein in signal transduction pathways involved in the positioning of developing neurons. Moreover, it was noted that the deficiency of either Reelin or both VLDLR and ApoER2 resulted in hyper-phosphorylation of the microtubule-associated TAU protein [19]. Interestingly, Reelin is more readily bound to ApoER2 compared to VLDLR. However, Reelin-induced DAB1 phosphorylation was fully eradicated in knockout *ApoER2*^−/−^ and *Vldlr*^−/−^ mice [67]. These outcomes display the critical roles of the LDLR family in the brain, particularly in neurodevelopment. Interestingly, expression levels of LDLR(s), such as LRP1, are also regulated by cholesterol levels [68]. As a result, it is important to maintain a proper level of brain cholesterol.

To balance cholesterol content in the brain, the excess cholesterol content needs to be excreted through the BBB in the form of 24S-hydroxycholesterol, which is a brain-specific oxysterol. The formation of 24S-hydroxycholesterol is facilitated *via* the activity of cholesterol-24S-hydroxylase in neurons, also known as cytochrome P450 family 46 subfamily A member 1 (CYP46A1) [21]. In the brain, this enzyme is expressed at a level about 100-fold higher than in the liver [22]. Moreover, in humans, approximately 80% of total 24S-hydroxycholesterol is detected in the brain compared to other organs [69]. Accordingly, the presence of 24S-hydroxycholesterol in blood circulation can be a marker of cholesterol homeostasis in the brain based on the ratio of 24S-hydroxycholesterol over cholesterol. A study using a 7-week-old knockout mouse model of the *Cyp46a1* gene reported an unaltered hepatic cholesterol metabolism in mutant mice compared to control mice. However, in the brain, de novo cholesterol synthesis was reduced by about 40% [22]. This study proposed that the excretion of cholesterol facilitated by CYP46A1 contributes to cholesterol turnover in the brain.

Overall, cholesterol is tightly regulated in the brain, accounting for many factors from transporters and receptors to cholesterol metabolism-associated enzymes (Figure 1). Therefore, impaired function of these important factors can lead to dysregulated cholesterol homeostasis, which may result in neurological complications.

### 2.3. Insights into Factors Involved in the Regulation of Brain Cholesterol Homeostasis

The homeostatic pool of cholesterol in the brain appears to be stable through excretion and de novo synthesis, as the plasma cholesterol cannot cross the BBB. In the adult brain, cholesterol is mainly synthesized in astrocytes. Likewise, astrocytes mainly create apolipoproteins, such as ApoE4 to transport cholesterol to neurons [70]. After exporting out of astrocytes, the ApoE-cholesterol complex enters the neurons by LDLR, which is then hydrolyzed by specific enzymes in the lysosome/endosome to release lipids/cholesterol. Subsequently, the Niemann–Pick C1 (NPC1) and Niemann–Pick C2 (NPC2) proteins release cholesterol to subcellular organelles for biological functions [23,24,71]. The redundant intracellular cholesterol in neurons is converted to 24S-hydroxycholesterol by the enzyme CYP46A1, which is then excreted by crossing the BBB. In the meantime, 24S-hydroxycholesterol also signals to astrocytes to induce the expression of ApoE so that the ApoE-mediated efflux of cholesterol from astrocytes to neurons is coordinated to maintain the pool of cholesterol in neurons [26]. Overall, brain cholesterol is securely regulated, whereby mutations in genes related to brain cholesterol metabolism can result in an imbalance in cholesterol homeostasis. Amongst these, impaired CYP46A1 and NPC1/2 functions have been extensively investigated.

A 2015 study showed that inhibition of CYP46A1 expression in hippocampal neurons led to significant accumulation of cholesterol. The authors reported a severe progressive neuronal loss followed by hippocampal atrophy and cognitive impairment as AD-like phenotypes. Moreover, the levels of phosphorylated TAU and the toxic Aβ40/42 peptides were abundantly elevated, suggesting that cholesterol accumulation in neurons may induce amyloid pathophysiology [8]. Two years later, the same group proposed that the overload of cholesterol in neurons disrupted lipid metabolism, such as sphingolipids (e.g., ceremide and glucosylceramide), as well as dampened lysosomal/endosomal trafficking [25]. Sphingolipids are important to the structure of the cellular membrane by supporting membrane integrity, fluidity, and signaling regulation [72].

Another cause of cholesterol accumulation in the brain is the well-known mutations in *NPC1/NPC2* genes. *NPC1* mutations cause 95% of Niemann–Pick type C (NPC) cases, while *NPC2* accounts for the remaining cases of the disease. NPC is an inherited autosomal recessive disorder, resulting in abnormal endosomal-lysosomal cholesterol trafficking [73]. NPC1 is located in the membrane of lysosomes and endosomes, acting to export cholesterol out of these organelles, while NPC2 helps transferring cholesterol to NPC1 [24,71]. Interestingly, beyond the investigation of mutated NPC1/2 related to cholesterol metabolism, studies also showed a correlation between impaired NPC1/2 and AD dementia [27,74].

Thus, the brain cholesterol is critically controlled by the local de novo synthesis, cholesterol transporters, lipoprotein receptors, and lysosomal enzymatic activity, to the excretion of redundant cholesterol (Figure 1). In this regard, it is also important to understand the function of cholesterol in neurons to appreciate how the brain cells maintain steady-state conditions of cholesterol.

## 3. Brain Cholesterol: Beyond the General Roles

Cholesterol plays a critical role in the human body, particularly in the brain. In addition to general roles, such as being a structural component in the cellular membrane, a precursor of sterol hormones, and vitamin D, as well as an essential component of bile acids, cholesterol and its derived metabolites are involved in many crucial biological processes and functions of brain cells.

### 3.1. Cholesterol as a Post-Translational Modification

Cholesterol molecules can be covalently attached to certain proteins as a result of post-translational modification to modulate the biological function of these proteins. For example, the typical candidate for this modification is the Hedgehog (Hh) protein family members (Figure 1), which are categorized into five subgroups: Indian hedgehog, Sonic hedgehog (Shh), Desert hedgehog, Echidna hedgehog, and Tiggy-winkle hedgehog [75,76]. After synthesis, the 46-kDa precursor Hh automatically undergoes an internal cleavage to produce a 19-kDa N-terminal segment and a 27-kDa C-terminal fragment [77]. Subsequently, cholesterol becomes attached to the 19-kDa N-terminal fragment at its C-terminal end [28]. The addition of cholesterol helps this fragment embed into specific sites of the cell membrane to further regulate the signaling cascade [78,79].

Studies have shown that in cholesterol-deficient conditions, the internal auto-processing is inhibited, resulting in a reduced mature Hh level and the accumulation of precursor forms, along with rapid degradation of both the mature and precursor forms [80]. Importantly, cholesterol modification of Hh is essential for a wide range of signaling cascades [29,81]. Accordingly, truncated Shh that lacks the site that binds to cholesterol is associated with malformations of different organs, including forelimb and hindlimb embryonic day (E) 18.5-day in mice. This study suggested that deficiency in Shh-cholesterol binding causes suppression of the heart- and neural crest derivatives-expressed (*dHand*) and fibroblast growth factor 4 (*Fgf4*) genes [29]. It is noted that dHAND and FGF4 proteins critically contribute to limb development during embryogenesis [82,83]. Similarly, other studies have suggested that properly activated Hh signaling is essential for embryogenesis, particularly in CNS development [30,31]. It has also been shown that Hh signaling is critical for the formation of other organs, such as bones, joints, and limbs [29,32,84,85,86]. However, dysregulated Shh signaling is associated with medulloblastoma (MB) brain tumors [33,87]. Therefore, dysregulated cholesterol levels, especially in the CNS, may lead to abnormal embryonic development and increase the risk of brain tumor formation, especially when associated with impaired regulation of the Hh family.

### 3.2. Cholesterol as an Important Composition of Lipid Rafts in Brain Cells

Lipid rafts are located within the bilayer of cell membranes, functioning as a microdomain platform to maintain membrane fluidity, regulate membrane trafficking (including endocytosis and exocytosis), and signal transduction. Lipid rafts are mainly made up of cholesterol and sphingolipids [6,34,35,88]. Impaired lipid rafts are associated with neurological disorders, including Alzheimer’s disease, Parkinson’s disease, and Huntington’s disease [36,38,39,40] (Figure 1).

The transmembrane amyloid precursor protein (APP) is highly expressed in brain cells that have a high metabolism to become available for neuronal synaptic transmission and plasticity [89,90]. As such, APP undergoes a series of catalytically proteolytic reactions through secretase enzymes, including α-secretase, β-secretase, and γ-secretase. The catalytic activity of α-secretase cleaves APP at the middle of the β-amyloid domain, which is non-toxically amyloidogenic to the brain. However, the cleavage activity of β-secretase and γ-secretase produces neurotoxic accumulation of Aβ peptides, which subsequently dampens synaptic transmission and neuronal plasticity. The toxic Aβ40/42 peptides contain 40 and 42 amino acids, respectively; there are two additional hydrophobic amino acids in Aβ42, which is also considered a major peptide present in the amyloid plaques of AD brains [91,92,93,94]. Recently, it has been suggested that therapeutic drugs that target γ-secretase may reduce the risk of toxic amyloidogenic processing [95]. Remarkably, studies have also recommended that abnormal cholesterol levels in lipid rafts significantly relate to the toxic Aβ production that is associated with γ-secretase activity [96,97,98,99]. As such, Urano and colleagues reported that statin treatment in neuroblastoma SH-SY5Y cells leads to a reduction of toxic Aβ production through the dissociation of γ-secretase from the lipid rafts [99]. An independent study showed abnormal cholesterol levels in post-mortem AD brains. The authors proposed that increased cholesterol may induce APP localization in lipid rafts, which may further promote the accessibility of APP to β-secretase and γ-secretase [96]. A recent study indicated that cholesterol synthesis inhibition in astrocytes greatly reduces the burden of toxic Aβ and tauopathy in an AD mouse model. Surprisingly, they also reported that, in conditions of low astrocyte-derived cholesterol levels, APP was prone to interact with α-secretase to generate soluble APP-α, which acts as a protective factor for neurons and is involved in many important biological processes in the brain [100]. Overall, evidence suggests that abnormally elevated cholesterol levels may be associated with the risk of Alzheimer’s disease. Thus, therapeutic approaches towards the control of dysregulated brain cholesterol may become promising for patients with AD.

Cholesterol levels are high in synaptic membranes and promote synaptic transmission by interacting with neurotransmitter receptors and modulating the expression of synapse-related proteins. Studies have shown that abnormal cholesterol levels may contribute to the aggregation of α-Synuclein, facilitating the interaction of membranes with α-Synuclein oligomers, which may disrupt cellular membrane integrity [101]. In this regard, α-Synuclein is a lipid-binding protein that is located in presynaptic terminals and is involved in the assembly of the soluble N-ethylmaleimide-sensitive-factor attachment protein receptor (SNARE) [102]. SNARE is an important protein complex in presynaptic neurons, responsible for the fusion of synaptic vesicles with the presynaptic membrane and promoting neurotransmitter exocytosis. Specifically, α-Synuclein is highly involved in PD pathophysiology [103]. A few years ago, scientists found that cholesterol is greatly involved in facilitating α-Synuclein binding to the surface of synaptic-like membranes, implying that excessive cholesterol may lead to α-Synuclein aggregation on the membranes, which may mechanically explain the role of cholesterol/α-Synuclein in PD [104].

Overall, in the brain, cholesterol significantly contributes to a wide range of biological processes, including post-translational modifications and the essential composition of lipid rafts. These biological functions are strictly maintained by a tight regulation of brain cholesterol. For this reason, abnormal levels of cholesterol may lead to many brain complications, such as Alzheimer’s disease, Parkinson’s disease, Huntington’s disease, and brain tumor(s). Abnormal cholesterol levels should be considered for treatment to prevent further severe problems. As such, therapeutic medications that can treat impaired brain cholesterol levels have been extensively investigated. Amongst cholesterol-lowering drugs, such as statins, bile acid sequestrants, niacin, fibrates, and others, statins have become the most well-tolerated and prescribed drugs.

## 4. Simvastatin: A Member of the Statin Family of Cholesterol-Lowering Drugs

Simvastatin (Zocor), a member of the statin family, is one of the top-used medicines in the world, used to lower elevated plasma cholesterol levels and treat cardiovascular diseases [105]. Simvastatin has also been studied as a potential treatment for different types of brain tumors and other brain complications [106,107,108,109,110]. Globally, extensive studies and clinical trials show the potential beneficial effects of simvastatin for different diseases. Herein, we will provide further details for simvastatin in terms of its mechanism of action, pharmacokinetics, pharmacodynamics, perspective history, and clinical trials.

### 4.1. Overview of Statins

**Figure 2:**
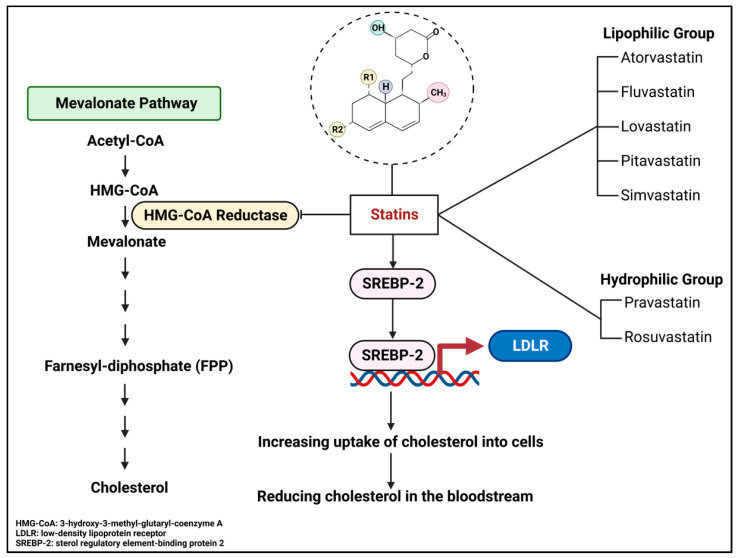
The natural structure, along with the function and different statin categories. The natural structure of statins includes a hexahydronaphthalene ring system and a polyketide structure. R1 and R2 are replaced by specific functional groups, according to different statins. Statins inhibit HMG-CoA reductase, a rate-limiting enzyme in the mevalonate pathway, which is responsible for producing cholesterol in the liver. Statins also promote SREBP-2 expression, which functions as a transcription factor for many genes related to lipid homeostasis regulation, including LDLR. LDLR presence on the membranes increases cholesterol absorption into the hepatocytes and other cells, helping to reduce plasma cholesterol levels. Information obtained from [111,112,119,120,121,122]. This Figure is created with BioRender.com.

Simvastatin (Zocor) is a member of the statin family of cholesterol-lowering prescribed medications. Based on their solubility, statins are categorized into two major groups: lipophilic and hydrophilic. The lipophilic group includes atorvastatin, fluvastatin, lovastatin, pitavastatin, and simvastatin, while the hydrophilic statins consist of pravastatin and rosuvastatin [111,112] (Figure 2). Statins are highly recommended for people with/at high risk of cardiovascular diseases and other vascular conditions, such as myocardial infarction, stroke, and atherosclerosis [113]. This is because statins mainly act as cholesterol-lowering drugs by inhibiting the activity of endogenous HMG-CoA reductase. The HMG-CoA reductase enzyme plays key roles in the mevalonate pathway by converting HMG-CoA to mevalonate (Figure 2), which is the intermediate step for many hydrophobic molecules, such as cholesterol, non-sterol isoprenoids, and sterol hormones [112,113].

Multiple clinical trials in the early 1990s indicated that lovastatin, pravastatin, and atorvastatin greatly reduced the level of LDL cholesterol in patients with familial combined hyperlipidemia (an inherited condition of primary dyslipidemia) and hypercholesterolemia [114,115,116]. For instance, in 1995, a 6-week double-blind clinical trial showcased the considerable atorvastatin-induced dose-dependent reduction in LDL cholesterol by 25% and up to 61% (25%, 29%, 41%, 44%, 50%, and 61%, corresponding to 2.5 mg, 5 mg, 10 mg, 20 mg, 40 mg, and 80 mg of atorvastatin, respectively) in outpatients with primary hypercholesterolemia compared to the baseline [116]. Since then, statins have become the primary option for treating dyslipidemia and hypercholesterolemia compared to other available lipid/cholesterol-modifying drugs, such as bile acid-binding resins (e.g., Cholestyramine), fibrates (e.g., Bezafibrate), and cholesterol-absorption inhibitors (e.g., Ezetimibe). This is supported by a national cohort study that used health databases in Alberta (Canada) to report that statins are one of the most common drugs recommended for patients with a high risk of cardiovascular diseases. Statins also accounted for 92.4% of total prescribed lipid-lowering drugs compared to other lipid-lowering drugs during the period of 2012 to 2016 [117]. Interestingly, amongst its two groups, lipophilic statins have a higher ability to enter the liver and extrahepatic tissues through passive diffusion and crossing the BBB. Of these, simvastatin has the highest lipophilicity [118], which is potentially beneficial to control abnormal cholesterol levels and other lipid-related pathways in the brain.

### 4.2. Historical Perspectives and Clinical Trials of Simvastatin

In December 1976, Akira Endo announced the investigation of new inhibitors for cholesterol synthesis. Isolation of three metabolite products produced by *Penicillium Citrinum* (ML-236A, ML-236B, and ML-236C) offered biological benefits that included reduction of cholesterol levels in the liver and serum of rats *via* oral administration. Endo and colleagues also examined the acute toxicity of these three agents in mice, employing 500 mg/kg by intraperitoneal injection and 2000 mg/kg through oral administration [123]. Subsequently, ML-236B was named mevastatin (or compactin). Four years later, mevinolin was successfully isolated from *Aspergillus terreus* by Alberts and colleagues [124], and it was named lovastatin. In the following years, simvastatin was semi-synthetically derived from lovastatin [125], which was originally named synvinolin. Since then, simvastatin’s effectiveness has been proven by different studies. For example, a double-blind, randomized study conducted in the UK in 2010 showcased the extensive reduction of LDL cholesterol when using simvastatin in doses of 20 mg or 80 mg in patients with a history of myocardial infarction [126].

Historically, two large-scale clinical trials entailed the beneficial effects of simvastatin on patients with cardiovascular diseases (CVD). In the Scandinavian Simvastatin Survival Study (4S) in 1994 in Scandinavian countries and the Heart Protection Study (HPS) in 2002 in the UK, an explicit reduction in mortality and morbidity of patients with coronary heart disease and all-cause vascular deaths was reported, respectively [127,128]. In addition to its therapeutic effects on cardiovascular diseases, simvastatin may also benefit other diseases, such as leukemia, esophageal cancer, pancreatic cancer, and prostate cancer, among others. [129]. Importantly, clinical trials of simvastatin for multiple diseases are conducted around the world for breast cancer (NCT03324425, NCT02096588), neurotoxicity (NCT04514029), Parkinson’s disease (NCT02787590), Alzheimer’s disease (NCT00053599), Smith-Lemli-Opitz syndrome (NCT00064792), sickle cell disease (NCT01702246), and tuberous sclerosis complex (NCT02061397).

### 4.3. Mechanism of Action, Pharmacokinetics, and Pharmacodynamics

**Figure 3:**
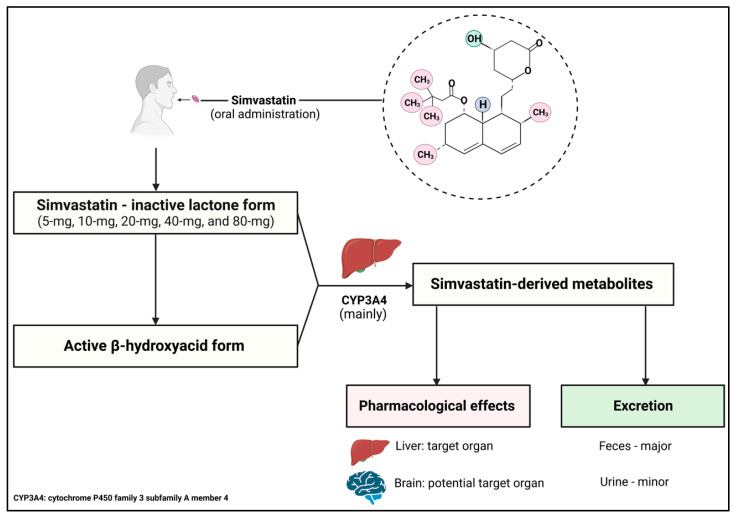
Pharmacodynamics and pharmacokinetics of simvastatin. Different doses of simvastatin (5 mg, 10 mg, 20 mg, 40 mg, and 80 mg) are available in the markets. To function in the body, simvastatin must be converted from the inactive lactone form into the active β-hydroxyacid form. Simvastatin metabolism mainly occurs in the liver through the main enzyme CYP3A4 to reach the proper concentration in the circulation. Simvastatin-derived metabolites are commonly excreted through the feces and in a minor amount in the urine. Information obtained from [125,138,139]. This Figure is created with BioRender.com.

As other statins, simvastatin is an effective therapeutic agent for mediating abnormally elevated plasma cholesterol. As such, the hydroxyglutaric acid component largely represents the pharmacophore of simvastatin. This component is similar to the structure of HMG-CoA, thus allowing the statin to competitively bind onto the active site of HMG-CoA reductase, leading to the inhibition of its enzymatic activity [130,131]. Endogenous HMG-CoA reductase is typically known as the rate-limiting enzyme in cholesterol synthesis (Figure 2). The basic structure of stains, shown in Figure 2, is chemically modified in the R1 and R2 positions to synthesize simvastatin. The R1 position is replaced by a 2,2-dimethylbutyrate ester group, which is very lipophilic, and R2 is replaced by a methyl group [125] (Figure 3). The 2,2-dimethylbutyrate ester group may subsequently help to promote increased CNS penetration of simvastatin. Interestingly, simvastatin also targets the expression of LDLR(s). As simvastatin inhibits cholesterol production in the periportal hepatocytes, a compensatory mechanism initiates an increase in LDLR(s) expression (Figure 2). Consequently, LDLR(s) on cell surfaces promote(s) the uptake of LDL cholesterol from the bloodstream to compensate the cholesterol content for cellular functions. Defects in LDLR(s) may lead to hypercholesterolemia. For example, familial hypercholesterolemia is a typical result of the dysfunctional LDLR [132]. Intriguingly, the increased simvastatin-induced LDLR(s) expression is facilitated by sterol regulatory element-binding protein 2 (SREBP-2). Studies have shown that simvastatin induces SREBP-2 expression, paralleled with a significant increase in HMG-CoA reductase and LDLR expression [119,133]. This suggests that simvastatin is capable of reducing cholesterol levels by removing cholesterol from the bloodstream and reducing cholesterol production in the liver (Figure 2). Additionally, simvastatin also offers pleiotropic properties for preventing platelet aggregation, inflammation, and endothelial dysfunction [134].

Due to the potential therapeutic application of simvastatin in different conditions, this drug has also been formulated to enhance its absorption in the gastrointestinal tract and its bioavailability in target organs. In this regard, scientists have developed a formulation of high-density lipoprotein nanoparticles (HDL NPs) with simvastatin, followed by a scalable method called “microfluidics” to create a uniform size of simvastatin-HDL NPs to reach an effective concentration in tumor cells. To examine the effect of simvastatin-HDL NPs on increasing the radiosensitivity of UM-SCC-1 cells, scientists have used UM-SCC-1 cells as a head and neck squamous carcinoma cell line. In such a case, simvastatin functions as a radiosensitizer that increases radiosensitization of tumor cells/tissues. This may be considered as a management for radiotherapy for cancers, particularly solid tumors. The authors suggested that this approach may reduce the side effects of high-dose radiotherapy that are commonly seen in cancer patients [135]. In another study, simvastatin was formulated as a form of nanoparticle dry powder inhalation used to treat pulmonary arterial hypertension. The idea is that this formulation can be optimized to a respirable size, which may effectively deposit simvastatin in the lungs [136]. Similarly, the powder inhaler formulation of simvastatin is proposed as a potential treatment for respiratory diseases due to its anti-inflammatory effects [137].

In principle, pharmacodynamics and pharmacokinetics are used to analyze the therapeutic effectiveness (dose-effect relationship) of drugs to minimize the side effects at certain doses. Pharmacokinetics is the study of drug processing in the body, which includes absorption, distribution, metabolism, and excretion, while pharmacodynamics aims to clarify the biochemical and pharmacological effects of drugs on specific target organs. Therefore, understanding these two principles can help improve beneficial therapeutic effects and reduce medication toxicity. Simvastatin has been clinically approved for oral administration and is available in 5 mg, 10 mg, 20 mg, 40 mg, and 80 mg tablets as an inactive lactone agent. The inactive form requires hydrolyzation by certain enzymes, such as carboxyesterase (plasma, liver, and intestine) and paraoxonase (serum), to convert it into the active form, β-hydroxyacid, which is a potent agent to suppress HMG-CoA reductase activity [138].

In general, the liver is the first-pass organ that simvastatin encounters through passive diffusion into the hepatocyte membranes. Simvastatin is then metabolized by cytochrome P450 family 3 subfamily A member 4 (CYP3A4) to reach the appropriate active dose in the body [139]. Simvastatin availability in circulation is normally about 5% [138,139]. Simvastatin has the highest lipophilicity of 4.68 amongst statins, which is measured by an octanol-water partition ratio, compared to other lipophilic statins [118,139]. Hence, simvastatin exhibits the highest capacity for crossing the BBB. The simvastatin-derived metabolites catalyzed by CYP3A4 also promote drug excretion. In the total amount of simvastatin and its metabolites excreted, fecal elimination accounts for 58% (bound to bile acids) and 13% by urination (Figure 3). Other regulatory aspects of simvastatin include its half-life of around 2–3 h. The maintenance of its bioavailability prior to deterioration and/or excretion could be attributed to plasma distribution, predominantly facilitated by its protein carriers, which account for 94–98% of its total plasma availability [139].

### 4.4. Drug Interaction of Simvastatin and Potential Side Effects

Simvastatin metabolism essentially occurs in the liver through a variety of enzymatic activities. In this regard, CYP3A4 plays an important role in mediating its toxicity [140]. Therefore, simvastatin is contraindicated with CYP3A4 inhibitors, such as HIV protease inhibitors, clarithromycin, ketoconazole, itraconazole, verapamil, diltiazem, and grapefruit juice [141,142]. This is important as there have been reported side effects of simvastatin despite its medicinal benefits; these include headaches and gastrointestinal issues (i.e., abdominal pain and constipation) [143,144]. The excess level of simvastatin in the plasma due to the presence of CYP3A4 inhibitors may also lead to detrimental complications, such as myopathy/rhabdomyolysis, a condition involving damaged skeletal muscle [145]. This may result from a reduction in simvastatin metabolism and elimination, which is normally metabolized by CYP3A4.

In general, statins are well tolerated; however, it has been reported that side effects can be observed in patients using high-dose statins for a certain period of time. The “prediction of muscular risk in observational conditions” study in France showcased that the onset of symptoms commonly occurred in 10.5% of the hyperlipidemic patients within one month after taking high-dose statins. Of these patients, 38% reported that muscle-related conditions, such as pain, heaviness, and cramps disrupted their daily activities [146]. Several mechanisms are suggested to be underlying these issues, such as mitochondrial impairments, coenzyme Q10 depletion, calcium signaling disruption, and vitamin D deficiency [147]. Remarkably, inactive lactone forms (simvastatin and lovastatin) provoked more myotoxicity compared to the active forms (atorvastatin, fluvastatin, pravastatin, rosuvastatin, and pitavastatin) [148]. Therefore, while statins, and in particular simvastatin, have demonstrated potent therapeutic applications, further studies could investigate the interactive risks with other medicines and/or pharmacological mechanisms that could cause secondary complications for patients.

## 5. Potential Therapeutic Application of Simvastatin for Brain Complications

**Figure 4:**
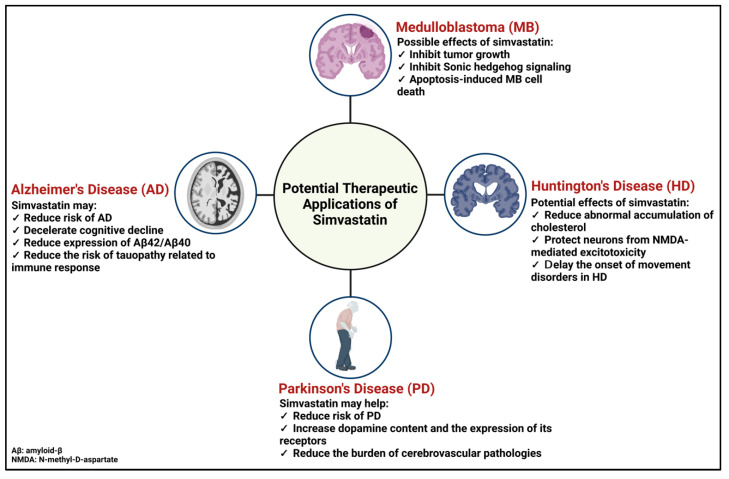
Potential applications of simvastatin for brain complications. Simvastatin may reduce the risk of Alzheimer’s disease and Parkinson’s disease as well as delay the onset of movement disorders in Huntington’s disease. Moreover, simvastatin may inhibit the growth of medulloblastoma. Information obtained from [13,107,156,160,164,165,166,167,168,169,170]. This Figure is created with BioRender.com.

Beyond functioning as a cholesterol-lowering agent, simvastatin may be a therapeutic option for brain complications, such as cerebral ischemia, intracerebral hemorrhage, and cerebral aneurysm [129,149]. In vivo studies and clinical trials of simvastatin applications for different diseases are shown in Table 1 and Table 2. Moreover, extensive studies have clearly indicated the link between abnormal cholesterol metabolism and brain complications, such as medulloblastoma [150,151], Alzheimer’s disease [152], Parkinson’s disease [153,154], and Huntington’s disease [155]. In this regard, many studies have shown the potential therapeutic application of statins, especially simvastatin, which has the highest lipophilicity, in brain complications associated with cholesterol abnormalities (Figure 4).

**Table 1:** In vivo studies of simvastatin treatment on brain complications.

Condition	Model	Dosage	Result
Medulloblastoma [156]	*Ptch1^+/−^* mice	40 mg/kg of body weight daily Duration: 2 weeks	Reduced size and growth rate of the tumor
Alzheimer’s disease [13]	Adult male guinea pigs	0.5% of diet (diet ≈ 25 g/day) Duration: 3 weeks	Reduced levels of Aβ42 and Aβ40 in the cerebrospinal fluid and in the brain
Huntington’s disease [157]	Male Wistar rats (6 µmol of intrastriatal administration of malonic acid to induce Huntington-like symptoms)	30 mg/kg of body weight daily Duration: 14 days	Significantly alleviated Huntington-like symptoms Restored mitochondrial activities Reduced neuro-inflammation (TNF-α and IL-6)

**Table 2:** Clinical trials of simvastatin treatment for brain complications.

Condition	Model	Dosage	Result
Secondary progressive multiple sclerosis NCT00647348 [158,159]	140 participants 18 to 65 years old Females and males Randomized, placebo-controlled	80 mg daily Duration: 24 months	Phase 2 Well tolerated and safe Reduced annualized whole-brain atrophy rate. Improved frontal lobe function and physical quality of life
Alzheimer’s disease NCT00303277 [160]	35 participants 18 to 90 years Females and males Randomized, controlled	40 mg daily Duration: 12 weeks	Phase 4 Reduced cerebrospinal fluid level of phospho-tau-181
Migraine NCT01225263 [161]	89 participants 18 Years and older Females and males Randomized, double-blind, placebo-controlled	20 mg, twice daily Dietary supplement: Vitamin D3, 1000 IU, twice daily Duration: 24 weeks	Phase 2 Effectively prevents episodic migraine
Stroke (acute) NCT01073007 [162]	104 participants 18 years and older Females and males Multicentric, randomized, double-blind	40 mg within 12 h from the onset Duration: 3 months	Phase 4, Decreased rate of bleeding complications Major neurological recovery
Subarachnoid hemorrhage-induced vasospasm NCT00235963 [163]	104 participants 18 years and older Females and males Randomized, double-blind, placebo-controlled	80 mg/day Duration: whether until discharge from the intensive care unit or a maximum of 21 days	Phase 1 Phase 2 Delayed cerebral ischemia

[NCT00647348]—https://clinicaltrials.gov/ct2/show/NCT00647348?term=simvastatin&recrs=e&draw=3&rank=101; [NCT00303277]—https://clinicaltrials.gov/ct2/show/NCT00303277?term=simvastatin&recrs=e&draw=4&rank=253; [NCT01225263]—https://clinicaltrials.gov/ct2/show/NCT01225263?term=simvastatin&recrs=e&draw=4&rank=247; [NCT01073007]—https://www.clinicaltrials.gov/ct2/show/NCT01073007; [NCT00235963]—https://clinicaltrials.gov/ct2/show/NCT00235963?term=simvastatin&recrs=e&draw=3&rank=185.

### 5.1. *Medulloblastoma*

Medulloblastoma is a cancerous brain tumor that normally appears in young children and during adulthood prior to the age of 40 [171,172]. MB mainly refers to a malignant pediatric tumor that develops in the cerebellum, a specific region in the back of the brain [173]. The name “medulloblastoma” was introduced in 1925 by Dr. Bailey and Dr. Cushing [174]. There are four subgroups of this cancer based on somatic mutations in certain genes and clinical symptoms: wingless (Wnt), sonic hedgehog (Shh), group 3, and group 4 [172,175]. The characteristics of groups 3 and 4 remain unclear. In general, the clinical symptoms include lethargy, nausea and vomiting, morning and evening headaches, anorexia (eating disorder), truncal ataxia, and abnormal gait and behavior [176]. In addition to the current treatments, such as tumor removal surgery, radiation therapy, and chemotherapy, targeted drug therapy has also emerged, particularly in the Shh group. The Shh subgroup of MB is the most well-characterized, where it accounts for about 28–30% of MB cases [177]. Importantly, the Shh pathway is essential to embryonic development in mammals. More importantly, the Shh-associated signaling cascades play critical roles in brain development that contribute to the establishment of distinct brain regions as well as the formation of neurons and glial cells [178]. Additionally, it contributes to hair and skin formation as well as limb development [179,180,181,182]. So, aberrant Shh signaling pathways can lead to many brain complications, for example, MB.

One study proposed that abnormal activation of Shh is sufficient to stimulate Notch pathway signaling, further targeting certain genes associated with proliferation and survival of MB cells [183]. Interestingly, sterol synthesis is important for the activity of Shh pathway signaling, particularly in cholesterol, and its derived oxysterols (OHC), such as 25S-OHC and 20S-OHC, which can subsequently stimulate the proliferation of MB cells [28,150,184]. Hence, it is believed that cholesterol homeostasis can become a potential target to suppress the Shh pathway-associated MB pathophysiology.

Recently, many studies have proposed the potential therapeutic options of simvastatin for medulloblastoma. It is suggested that simvastatin can inhibit medulloblastoma brain tumor growth by inhibiting cancer cell proliferation and reducing activation of hedgehog signaling [156,164]. Importantly, the researchers also found that simvastatin did not cause any negative effects on bone growth in tumor-bearing mice due to the fact that cholesterol biosynthesis and Shh signaling cascades are critical to bone development [164]. In 2019, our team reported apoptosis-induced medulloblastoma cell death mechanisms. We used three different MB cell lines, such as Daoy, D283, and D341 (from the SHH subgroup, subgroup 3/4, and subgroup 3, respectively), to investigate the molecular effects of simvastatin on medulloblastoma cells. Our results showed that the simvastatin apoptosis-induced cell death was dose-dependent in all three cell lines. Moreover, an induction of caspase-dependent apoptosis in terms of caspases 3/7, 8, and 9 was noticed. Simvastatin altered the expression level of certain apoptosis-related factors, such as Bax, Bcl-2, and Bcl-xl. This study thus highlighted the potential application of simvastatin as a novel non-classical adjuvant therapy for MB [107] (Figure 4). Similarly, in vivo studies also indicated the potential effects of simvastatin on suppressing MB growth in terms of a reduction in tumor volume and inhibiting Shh pathway-associated MB [156,164]. As a result, simvastatin may become a promising treatment for MB. In the Daoy Shh MB cells, simvastatin was also tested regarding the regulation of the methyl CpG-binding protein 2 (*MECP2*) gene [106]. The encoded MeCP2 protein is an epigenetic factor [185,186], with specific involvement in gene transcription, brain metabolism, and neurodevelopmental disorders, including Rett Syndrome [58,187,188,189,190,191,192]. However, more recently, MeCP2 has been reported to have oncogenic properties in certain types of human cancer [193].

### 5.2. Alzheimer’s Disease

Globally, Alzheimer’s disease accounts for 60–70% of dementia cases, impacting 33–38 million people worldwide (World Health Organization, 2022 https://www.who.int/news-room/fact-sheets/detail/dementia). The deposition of Aβ and the formation of hyperphosphorylated TAU are the main causes of Alzheimer’s disease [194]. Affected patients develop dementia, memory loss, cognitive difficulties, and an inability to perform their basic daily activities. There is currently no cure for Alzheimer’s disease, which places a greater emphasis on medical management. Extensive studies have established a strong association between abnormal cholesterol levels and Alzheimer’s disease. Impaired cholesterol metabolism and its derived metabolites, such as 24S-OHC, are associated with the development of hyperphosphorylated TAU and Aβ deposition [195,196,197]. Studies have also proposed that two essential factors for brain cholesterol metabolism (ApoE4 and CYP46A1) can become amenable targets for treating Alzheimer’s disease [198,199,200,201]. One study demonstrated that, amongst the major alleles of *APOE*, there is a significant association between *APOE4* and the late onset of Alzheimer’s disease [202], meaning that ApoE4 seeds Aβ plaque more abundantly compared to other ApoE members.

Importantly, a prospective study indicated that the use of statins, such as simvastatin and pravastatin, may reduce the risk of Alzheimer’s disease and other age-associated neurodegenerative disorders. In this study, the authors showed that histone deacetylase 2 (*HDAC2*) and integrin sub-unit alpha L (*ITGAL*) genes were targeted by simvastatin and lovastatin. HDAC2 is an epigenetic factor, while ITGAL contributes to cellular adhesion. By applying the systems biology approach, the authors noticed that simvastatin targeted *HDAC2* and *ITGAL* genes, which are further involved in lipid transport, biosynthesis of fatty acids, regulation of LDLR, and cellular response to Aβ. The study implied the molecular effects of simvastatin on epigenetic factors that may exhibit the potential impact of simvastatin on reducing the risk of Alzheimer’s disease [165]. Another study indicated that simvastatin may help decelerate the progression of cognitive decline in AD patients, especially in those who are *APOE4* homozygous [166]. Interestingly, simvastatin reduced the expression of Aβ42 and Aβ40 peptides in primary cultures of hippocampal neurons [13], as well as the level of phospho-TAU-181 in the cerebrospinal fluid [160]. A recent study indicated that tauopathy may lead to the development of an innate and adaptive immune response in the brain. This resulted in the degradation of microglia and T cells, which are critical in blocking the formation of tau-mediated neurodegenerative diseases, such as Alzheimer’s disease [203]. Studies have proposed the anti-inflammation of simvastatin in microglial cells in terms of a reduction in cytokine interleukin-1β (IL-1β) expression and activation of microglial cells after traumatic brain injury and neuroinflammation [204,205]. In short, simvastatin may provide promising therapeutic effects for patients with Alzheimer’s disease through a variety of mechanisms, from cholesterol-mediated effects to immune responses (Figure 4).

### 5.3. Parkinson’s Disease

Parkinson’s disease is a brain disorder with unknown causes. However, researchers have noticed within its pathophysiology that the basal ganglia, which controls voluntary movement, develops an impaired neural network. In addition, reduced levels of neurotransmitters, such as dopamine and serotonin, are found in PD patients [206]. Moreover, amyloid formation in the form of α-synuclein (Lewy bodies) is found in the brains of PD patients [207]. A recent study also found impaired lipid homeostasis in neurons, astrocytes, and microglia in the post-mortem brains of PD patients [208]. These findings may help explain unintended or uncontrollable movements and the potential mechanisms for Parkinson’s disease. As the disease advances, the affected patients may display trouble walking and talking. They may also experience mental problems, behavioral changes, and suffer from impaired memory while exhibiting sleep problems, and depression. In North America, Parkinson’s disease incidence is 572/100,000 of people over 45 years old, whilst also increasing in risk with ages 65 and above [209]. Despite the unknown causes, several studies have pointed towards an association between cholesterol metabolism and Parkinson’s disease [153,210,211], particularly the role of cholesterol in the pathophysiology of Lewy body [9]. Thus, it is suggested that controlling cholesterol levels may be a strategy to help prevent and reduce the risk of Parkinson’s disease.

Studies have shown that oral administration of 10mg/kg of body weight of simvastatin for 4 weeks in Sprague–Dawley rats restored the expression of D1 and D2 receptors for dopamine. The authors proposed that the molecular effect of simvastatin on inducing D1 and D2 expression may ameliorate PD symptoms [167]. Another study complemented this result by demonstrating that simvastatin (10mg/kg of body weight, a 4-week treatment) significantly increased dopamine content in the striatum [168]. Lately, scientists have believed that statins may also help reduce the risk of PD in older adults. This could be attributed to the use of lovastatin, simvastatin, and atorvastatin, which greatly reduced the severity of atherosclerosis and the burden of cerebrovascular pathologies that usually occur at later stages in life. As a result, this may lessen the incidence of Parkinsonism, which is an umbrella term for neurological disorders with Parkinson-like symptoms and movement conditions related to progressive loss of motor function [212] (Figure 4).

### 5.4. Huntington’s Disease

Huntington’s disease was named after George Huntington, who first reported the disease in 1872 [213]. HD is a progressively autosomal dominant neurodegenerative disorder caused by a mutation in the *Huntingtin* gene, which was first announced in 1993 [214]. This mutation results in the abnormal expansion of the CAG trinucleotides, which forms a gain-of-function protein and dampens certain brain region activity [215]. As a result, patients with Huntington’s disease suffer from movement disorders, dementia, changes in personality and behavior, as well as eating and speaking difficulties. Although it has been investigated for more than 150 years since its discovery, this disease is incurable, with a high incidence of 4.88/100,000 people per year (in Europe and North America) [216]. Therefore, it is essential to find effective treatments to alleviate HD symptoms.

In 2006, a group of researchers applied gas chromatography and time-of-flight-mass spectrometry to analyze metabolic profiling in both patients and HD mouse models. The result showed that certain intermediate products in lipid metabolism may act as a strong biomarker for the early onset of Huntington’s disease due to their significantly elevated levels [217]. It thus implies the importance of cholesterol metabolism in HD. In this regard, many studies focus on the application of statins to alleviate HD clinical symptoms. For example, a study showcased an accumulation of cholesterol and increased expression of Caveolin-1 in a HD mouse model, along with the induction of NMDA (N-methyl-D-aspartate)-mediated excitotoxicity. However, after simvastatin treatment, the authors noticed a reduction in both abnormal cholesterol and Caveolin-1 expression, along with protecting mutant *Huntingtin* striatal neurons from NMDA-mediated excitotoxicity [169]. Caveolin-1 functions as a structural protein that specifically binds to cholesterol in cell membranes and regulates intracellular cholesterol levels [218,219]. Meanwhile, the NMDA receptor is responsible for regulating the excitatory neurotransmitter glutamate [220].

With the same treatment, a group of researchers indicated that simvastatin improved body weight, balance beam walking performance from decreased slips, locomotor activity, and rotarod performance in male mevalonic acid-induced HD Wistar rats. Moreover, the authors noticed simvastatin reduced brain oxidative damage and restored the activity of most mitochondrial enzymes [157]. Additionally, a multicenter, longitudinal, and observational study in 2018 showcased that the statin family delayed the onset of motor symptoms in patients with HD. Simvastatin was the most used compared to atorvastatin, rosuvastatin, lovastatin, and pravastatin. The result showcased that after an average of 5.71 years, statin users had a significantly delayed onset of motor diagnosis [170]. Overall, the use of simvastatin may provide beneficial effects for Huntington’s disease, not only alleviating and delaying the clinical symptoms but also mediating excitotoxicity in Huntington’s disease (Figure 4).

## 6. Conclusions and Implications

Beyond functioning as a cholesterol-lowering agent, simvastatin may offer potential therapeutic effects for other diseases, particularly those that involve complications of the brain, where cholesterol metabolism is locally and strictly regulated. Abnormally elevated cholesterol levels are strongly associated with many brain complications and diseases, ranging from different types of cancer to neurodegenerative disorders. On these points, simvastatin may provide beneficial therapeutics for certain diseases; however, the functional significance of simvastatin for brain complications is still controversial. Despite the promising data and observations, further studies are needed to investigate the mechanistic insight of simvastatin toward neurological diseases, which are associated with impaired brain cholesterol metabolism.

In addition, researchers may consider the use of simvastatin as an optional treatment for neurodevelopmental disorders, which may also be relevant to the imbalanced brain metabolism. In agreement with the complexity of cholesterol metabolism in the brain as well as the limited treatment options for brain complications, simvastatin and its mechanism of action may become a promising therapeutic strategy for brain and neurological diseases, including brain tumors, neurodegenerative diseases, and neurodevelopmental disorders.

## Data Availability

Not applicable.

## Abbreviations

Aβ: amyloid β; AD: Alzheimer’s disease; ApoB: apolipoprotein B; ApoE: apolipoprotein E; ApoER2: apolipoprotein E receptor 2; APP: amyloid precursor protein; BBB: blood-brain barrier; CNS: central nervous system; CVD: cardiovascular diseases; CYP3A4: cytochrome P450 family 3 subfamily A member 4; CYP46A1: cytochrome P450 family 46 subfamily A member 1; DAB1: disabled-1; *dHand*/dHAND: heart- and neural crest derivatives-expressed protein; E: embryonic day; *Fgf4*/FGF4: fibroblast growth factor 4; FH: familial hypercholesterolemia; HD: Huntington’s disease; *HDAC2*/HDAC2: histone deacetylase 2; HDL: high-density lipoprotein; HDL NPs: high-density lipoprotein nanoparticles; Hh: hedgehog; HMG-CoA: 3-hydroxy-3-methylglutaryl coenzyme A; ITGAL/ITGAL: integrin sub-unit alpha L; LDL: low-density lipoprotein; LDLR: LDL receptor; LRP1: low-density lipoprotein receptor-related protein-1; MB: medulloblastoma; *MECP2*/MeCP2: methyl CpG-binding protein 2; NMDA: N-methyl-D-aspartate; NPC: Niemann–Pick type C; NPC1: Niemann–Pick C1; NPC2: Niemann–Pick C1; OHC: oxysterols; PD: Parkinson’s disease; PSD-95: postsynaptic density protein 95; Shh: sonic hedgehog; SNARE: soluble N-ethylmaleimide-sensitive-factor attachment protein receptor; SREBP-2: sterol regulatory element-binding protein 2; VLDL: very-low-density lipoprotein; VLDLR: very-low-density lipoprotein receptor.
